# Artificially decreased vapour pressure deficit in field conditions modifies foliar metabolite profiles in birch and aspen

**DOI:** 10.1093/jxb/erw219

**Published:** 2016-06-02

**Authors:** Jenna Lihavainen, Markku Keinänen, Sarita Keski-Saari, Sari Kontunen-Soppela, Anu Sõber, Elina Oksanen

**Affiliations:** ^1^University of Eastern Finland, Department of Environmental and Biological Sciences, PO Box 111, 80101 Joensuu, Finland; ^2^University of Tartu, Institute of Ecology and Earth Sciences, Lai 40, 51005 Tartu, Estonia

**Keywords:** *Betula*, GC-MS, metabolite profiling, mineral nutrients, *Populus*, relative humidity, VPD.

## Abstract

Relative air humidity (RH) is expected to increase in northern Europe due to climate change. Increasing RH reduces the difference of water vapour pressure deficit (VPD) between the leaf and the atmosphere, and affects the gas exchange of plants. Little is known about the effects of decreased VPD on plant metabolism, especially under field conditions. This study was conducted to determine the effects of artificially decreased VPD on silver birch (*Betula pendula* Roth.) and hybrid aspen (*Populus tremula* L.×*P. tremuloides* Michx.) foliar metabolite and nutrient profiles in a unique free air humidity manipulation (FAHM) field experiment during the fourth season of humidity manipulation, in 2011. Long-term exposure to decreased VPD modified nutrient homeostasis in tree leaves, as demonstrated by a lower N concentration and N:P ratio in aspen leaves, and higher Na concentration and lower K:Na ratio in the leaves of both species in decreased VPD than in ambient VPD. Decreased VPD caused a shift in foliar metabolite profiles of both species, affecting primary and secondary metabolites. Metabolic adjustment to decreased VPD included elevated levels of starch and heptulose sugars, sorbitol, hemiterpenoid and phenolic glycosides, and α-tocopherol. High levels of carbon reserves, phenolic compounds, and antioxidants under decreased VPD may modify plant resistance to environmental stresses emerging under changing climate.

## Highlight

Metabolite profiling and mineral nutrient analysis revealed that long-term exposure to decreased vapour pressure deficit (VPD) promotes production of starch, phenolic compounds, and antioxidants, and affects nutrient status in tree leaves.

## Introduction

Climate change scenarios predict an increase in temperature by 2.3–4.5 °C and in precipitation by 5–30% in the Baltic area by the year 2100 (Kont *et al.*, 2003). Atmospheric water content (specific humidity) will increase in the northern latitudes (IPCC, 2013), leading to cloud formation and affecting the distribution and intensity of precipitation (Willett *et al.*, 2007; Schneider *et al.*, 2010). It is expected that relative air humidity (RH) will increase in northern Europe due to increasing precipitation accompanied by increasing cloud cover and frequency of wet days. Increasing air humidity decreases evapotranspiration and modifies the water cycle between the biosphere and atmosphere.

Since leaf to air vapour pressure deficit (VPD) determines the diffusion of water vapour from plants, elevated RH reduces the evaporative demand and transpiration rate. Apart from climatic factors (temperature, RH, wind speed, and solar radiation), the transpiration rate is influenced by biological factors such as stomatal characteristics, canopy conductance, and canopy structure that affect the resistance to transpiration (Allen *et al.*, 1998). In addition, the transpiration rate is affected by leaf temperature, cuticle properties, plant and soil water status, and nutrient availability in the soil (Grantz, 1990; Cramer *et al.*, 2009; Matimati *et al.*, 2014). Leaf wetness occurs more frequently under high humidity, and the condensation of water droplets on the leaf surface disturbs CO_2_ exchange and photosynthesis (Ishibashi and Terashima, 1995; Brewer and Smith, 1997; Letts and Mulligan, 2005).

The transpiration rate has been shown to have a positive correlation with the mineral nutrient uptake of plants (McDonald *et al.*, 2002; Novák and Vidovik, 2003; Cramer *et al.*, 2009; Cernusak *et al.*, 2011). A reduced transpiration rate under low VPD may restrain nutrient uptake of plants by the mass flow of water, which brings water and nutrients in contact with the root system, enabling their uptake (Barber, 1962). However, at the same time, low VPD promotes stomatal opening, facilitating influx of CO_2_ to the leaf mesophyll which, in turn, can enhance carbon fixation (Bunce, 1984; Fordham *et al.*, 2001; Oren and Pataki, 2001). Stomata are sensitive to solar radiation, plant and soil water status, and to several other factors (reviewed in Buckley, 2005) along with VPD, which is an important factor mediating the stomatal response to CO_2_ (Talbott *et al.*, 2003).

Since plants need to adjust their carbon fixation to nutrient assimilation, a change in carbon or nutrient supply will require readjustment of leaf metabolism. If nitrogen (N) assimilation does not keep up with carbon fixation, the N concentration may decrease and carbohydrates may increase in the leaves. This is often observed when plants are grown under elevated atmospheric CO_2_, especially if N supply is low (Geiger *et al.*, 1999; Ainsworth and Long, 2005). Carbohydrates and especially starch accumulate in the leaves of N-limited plants (Paul and Driscoll, 1997; Schlüter *et al.*, 2013), whereas starch content may decline if phosporus (P) supply is low (Schlüter *et al.*, 2013). Shirke and Pathre (2004) studied the short-term effects of VPD on starch and sucrose content in potted *Prosopis juliflora* plants grown outdoors. They found that starch and sucrose levels were highest when leaves were exposed to a VPD of 3 kPa, and the levels declined when leaves were exposed to a VPD of above or below 3 kPa (Shirke and Pathre, 2004). In addition to carbon storage, the balance between photosynthetic carbon assimilation and nutrient metabolism can be achieved by directing excess carbon to carbon-based secondary metabolites, as is observed in response to low N and P supply (Koricheva *et al.*, 1998; Schlüter *et al.*, 2012, 2013) and under elevated atmospheric CO_2_ (Peñuelas *et al.*, 1997; Agrell *et al.*, 2000).

Apart from altered carbon and nutrient supply, trees need to acclimate to altered water availability in response to decreased VPD in the field. In free air humidity manipulation (FAHM) studies, humidification treatment has been shown to increase the local water availability in the soil indirectly when evapotranspiration is low (Parts *et al.*, 2013; Niglas *et al.*, 2014; Sellin *et al.*, 2014). It is a common feature in trees that they acclimate to soil water conditions by altering their primary metabolism, including changes in carbohydrate metabolism, and osmotic adjustment by compatible solutes (Kreuzwieser *et al.*, 2004; Jaeger *et al.*, 2009; Yang *et al.*, 2011; Kreuzwieser and Rennenberg, 2014). Oxidative stress occurs under adverse abiotic stress conditions, such as nutrient deficiency (Mittler, 2002), waterlogging, and hypoxia (Kreuzwieser *et al.*, 2004; Jaeger *et al.*, 2009; Possen *et al.*, 2011). Formation of reactive oxygen species (ROS) under oxidative pressure can be restrained by antioxidants, such as ascorbate (AsA), glutathione, tocopherols, and phenolic compounds. Together with antioxidant enzyme systems, these compounds would help to limit oxidative damage and maintain redox homeostasis of the cells.

In a controlled growth chamber study with young silver birch, we observed that short-term exposure to low VPD changed primary metabolism considerably in 26 d (Lihavainen *et al.*, accompanying manuscript). The levels of N-containing metabolites (amino acids and chlorophyll) and soluble carbohydrates were lower, whereas starch and flavonoid levels were higher in the leaves that were developed in low VPD than in high VPD (Lihavainen *et al.*, accompanying manuscript). Additional N supply mitigated the effect of low VPD on leaf metabolites, suggesting that the metabolic changes were elicited by N limitation.

The effects of artificially decreased VPD on two deciduous tree species, silver birch (*Betula pendula* Roth.) and hybrid aspen (*Populus tremula* L.×*P. tremuloides* Michx.), have been subject to investigation since 2007 in a long-term FAHM field experiment conducted in Estonia (Kupper *et al.*, 2011). Long-term exposure to decreased VPD reduced the water flux through a deciduous tree canopy (Kupper *et al.*, 2011) and the concentrations of N and P in birch and aspen leaves during the first three seasons of the FAHM experiment (Tullus *et al.*, 2012; Sellin *et al.*, 2013).

The objective of our study is to determine the changes in foliar metabolite profiles in silver birch and hybrid aspen in response to long-term exposure to decreased VPD in realistic field conditions during the fourth growing season. In theory, low VPD may benefit carbon gain, but restrain transpiration-driven nutrient uptake via mass flow of water. Therefore, humidification treatment is expected to affect the balance between carbon fixation and nutrient acquisition, which would be reflected in primary carbohydrate and N metabolism of tree leaves. Decreased VPD is hypothesized to increase starch and to decrease nutrient concentration in the tree leaves, N and P in particular, as was observed in previous study years. Because N and P deficiencies have contrasting effects on primary metabolites (Schlüter *et al.*, 2013), it is difficult to predict the metabolite responses to humidification treatment in the FAHM experiment. Typically the levels of metabolites containing the limiting element decreases; N-containing compounds, such as amino acids, chlorophyll, and proteins, decrease when N supply is low, and P-containing compounds, such as sugar phosphates, decrease when P supply is low (Schlüter *et al.*, 2013). A typical response to both N and P deficiencies is the accumulation of carbon-based secondary metabolites produced via the phenylpropanoid pathway (Koricheva *et al.*, 1998; Schlüter *et al.*, 2013). Therefore, we expect that decreased VPD would increase phenylpropanoid production.

Long-term field experiments face considerable challenges, as foliar metabolism is influenced by several factors, such as pests and diseases, soil factors, and weather conditions, making the interpretation of the results difficult. However, a field study gives a realistic view of VPD impacts on tree metabolism in natural conditions. Besides reduced transpiration-driven mass flow of water and nutrients, changes in soil processes and acclimation mechanisms are also likely to change the nutrient availability in the soil and nutrient uptake by trees in long-term humidification treatment (Hansen *et al.*, 2013; Parts *et al.*, 2013; Rosenvald *et al.*, 2014). Metabolite data are thus supplemented with mineral nutrient data. Environmental metabolomics has potential to detect metabolic responses to a combination of various environmental stressors, and the results from this kind of study can be used to generate further hypotheses to unravel the underlying mechanisms behind plant responses (Bundy *et al.*, 2009).

## Materials and methods

### Experimental area

The FAHM field site is located in the Järvselja Experimental Forest District in south eastern Estonia (58°14'N, 27°18'E), in the boreo-nemoral vegetation zone and in the humid continental temperate climate zone. Average annual precipitation in the area is 650mm, the mean temperature of the warmest month, July, is 17 °C, and the coldest is January at −6.7 °C. The growing season lasts from mid-April to October, ~175–180 d.

The experimental site was established in an abandoned agricultural area in 2006–2007. Trees were planted in spring (birch) and autumn (aspen) 2006. The experimental site comprises 14×14 m experimental plots, three replicated free air control plots with ambient air humidity and three humidified plots, where the RH was increased, on a long-term average by 7–8% over the ambient level. Concomitantly, humidification treatment decreased VPD by 26% compared with the ambient level (Tullus *et al.*, 2012). In 2011, soil water potential and soil pH were higher in the humidified plots than in the control plots (Parts *et al.*, 2013; Niglas *et al.*, 2014). In 2011 (June–August), VPD was on average 0.7–0.95 kPa and 0.85–1.15 kPa during afternoon hours (12:00–16:00h in humidified and control plots, respectively (Niglas *et al.*, 2014).

Each plot was split between two species: silver birch (*B. pendula* Roth.) seedlings (RMK Kullenga Nursery, Estonia) and micro-propagated hybrid aspen (*P. tremula* L.×*P. tremuloides* Michx.) (clone C05-99-35, Finnish Plant Production Inspection Centre). Tree density in the experimental plots was 10 000 trees ha^−1^. For a detailed description of the experimental field and technical details of the misting system, see Kupper *et al.* (2011) and Tullus *et al.* (2012).

### Sampling

Samples were collected during the fourth season of humidity manipulation, in 2011 (misting started in 2008). Six birch and six aspen trees were sampled from three control (ambient VPD) and three humidified experimental plots (decreased VPD) (*n*=18 for each species in each VPD treatment). Leaf samples for metabolite analysis were sampled at the beginning of July. One leaf disc (ø 1cm) was taken from four healthy-looking, intact, short shoot, sun leaves from four sides of the tree, around the middle of the canopy level. Since short shoot leaves are developed at the beginning of the growing season, the sampled leaves were the same age. The leaf discs were pooled and immediately frozen in liquid nitrogen, and stored at −70 °C. Ten leaves were collected from the same trees after the sampling of leaf discs. These leaves were dried, pressed, and used for leaf trait measurements and mineral nutrient analysis.

### Leaf traits and mineral nutrients

Leaf area and specific leaf area (SLA) were measured as an average of 10 leaves. Leaf area (cm^2^) was defined using LAMINA software (Bylesjö *et al.*, 2008). The SLA was measured as leaf area (cm^2^) divided by dry weight (g).

The dried leaves were milled (~0.5g) and the N concentration (mg g DW^−1^) was determined from 12 plants per treatment (four replicates from each experimental plot) by the Kjeldahl method. Other elements (B, Ca, Cd, Co, Cr, Fe, K, Mg, Mn, Na, Ni, P, S, V, and Zn) were determined from six plants per treatment (two replicates from each experimental plot) using an inductively coupled plasma optical emission spectrometer (ICP-OES). Leaf powder was dried at 105 °C prior to the analysis. The samples were digested by the standard EPA-3051 method: microwave digestion (MARS5) with nitric acid and water (6:1 HNO_3_:H_2_O), and measured using ICP-OES (IRIS Intrepid II XSP).

### Reagents

Labelled standards, benzoic-d_5_ acid, d-glucose-^13^C_6_, and glycerol-d_8_, were purchased from Campro (Germany) and dl-alanine-2,3,3,3-d_4_ from Isotec (USA). Methanol was purchased from Merck (Germany), DMSO from BDH Chemicals (UK), *N*-methyl-*N*-(trimethylsilyl)trifluoro-acetamide with 1% trimethylchlorosilane (MSTFA with 1% TMCS) from Thermo Scientific (USA), and sodium acetate from JT Baker (USA). The remaining chemicals used were obtained from Sigma (Germany).

### Metabolite extraction

The leaf disc samples (~50mg FW) were kept frozen with liquid nitrogen while homogenized in 2ml safe-lock Eppendorf tubes with a bead mill (15s, 20 Hz, with a 5mm stainless steel bead) (TissueLyser, Qiagen, Germany). The leaf powder was extracted in two steps: first with 1.0ml of 100% methanol and secondly with 1.0ml of 80% (v/v) aqueous methanol. During the first extraction step, internal standard mix solution (50 µl) was added to each sample (benzoic-d_5_ acid 0.309mg ml^−1^, d-glucose-^13^C_6_ 0.316mg ml^−1^, glycerol-d_8_ 0.200mg ml^−1^, 4-methylumbelliferone 0.768mg ml^−1^, dl-alanine-2,3,3,3-d_4_ 0.018mg ml^−1^ in DMSO). During both extraction steps, the samples were extracted at 4 °C for 15min at 1400rpm (Thermomixer, Eppendorf, Germany) and centrifuged at 10 °C for 3min at 13 000rpm (13 500 *g*). The supernatants were combined, and 100 µl (birch) or 80 µl (aspen) of the supernatant was dried in a vacuum. The vials with the dry residue were degassed with N, frozen, and kept overnight at −70 °C.

### Starch analysis

Starch was determined from the vacuum-dried plant residue (stored at −70 °C) after extraction of soluble metabolites. The pellets were homogenized in 1ml of water with a bead mill in 2ml safe-lock Eppendorf tubes (60s, 20 Hz, with a 5mm stainless steel bead) and diluted to 5ml in 10ml sample tubes. Part of the homogenate (0.5ml) was heated at 100 °C for 60min at 200rpm to gelatinize the starch. The starch was digested to glucose in a reaction comprising 0.5ml of the sample and 0.5ml of enzyme solution including 6U of α-amyloglucosidase and 1U of α-amylase in 200mM sodium acetate buffer (pH 4.8). The samples were incubated at 50 °C for 20h and enzyme activity was stopped by heating the samples at 100 °C for 2min. The samples were centrifuged at 10 °C for 5min at 13 000rpm (13 500 *g*), and glucose was determined from the supernatant by enzymatic assay with hexokinase and glucose 6-phosphate dehydrogenase (Glucose HK assay kit, Sigma).

### Metabolite profiling by gas chromatography–mass spectrometry

The frozen samples were allowed to reach room temperature before adding 20 µl of alkane series (C10–C30) solution in hexane as a retention time standard, and dried in a vacuum. The samples were then redissolved in 40 µl of methoxyamine hydrochloride (MAHC) (20mg ml^−1^) in pyridine and incubated for 180min at 30 °C at 200rpm. Finally, the samples were derivatized with 80 µl of MSTFA with 1% TMCS for 120min at 60 °C, at 200rpm.

The GC-MS system consisted of the Agilent 6890N gas chromatograph system, mass spectrometer (5973N), autosampler (7683), and injector (7983) (Agilent Technologies, Palo Alto, CA, USA). Split injection (2 µl) was employed, using a deactivated Split precision liner (Restek, Bellefonte, PA, USA) with a split ratio of 40:1. The column was 30 m Rxi-5Sil MS, 0.25mm ID, 0.25 µm df with 10 m Integra-Guard (Restek, Bellefonte, PA, USA). The injection temperature was set to 260 °C, MSD interface 290 °C, MS source 230 °C, and MS quad 150 °C. Helium (1.0ml min^−1^) was used as a carrier gas. The oven temperature program was as follows: 1min isothermal heating at 70 °C, followed by a 6 °C min^−1^ ramp to 330 °C, 6min at 330 °C, and a post-run at 70 °C for 3min. Mass spectra were collected at 2.94 scans s^–1^ with a scanning range of 55–550 *m/z*. In addition, a broader scanning range of 55–650 *m/z* was used for compound identification.

Deconvolution, component detection, and quantification were conducted with a Metabolite Detector (2.06 beta) (Hiller *et al.*, 2009), and co-eluting components were confirmed with AMDIS (version 2.66, NIST). The relative content of the metabolite was calculated as metabolite peak area normalized by the peak area of the internal standard, benzoic-d_5_ acid, and the fresh weight of the sample.

Altogether 196 compounds were detected from the birch and aspen leaves by untargeted GC-MS analysis (Supplementary Table S1 at *JXB* online). The birch and aspen data sets included 160 metabolites, of which 124 metabolites were abundant in both species. About half of the GC-MS metabolites were annotated on the basis of spectral data and retention index matched to the NIST Mass Spectral Database (version 2.2, Agilent Technologies), the Golm Metabolome Database (GMD) (Schauer *et al.*, 2005; Hummel *et al.*, 2010), and to standard compounds, when available. Salicinoids and a hemiterpenoid glycoside were tentatively annotated based on fragmentation patterns and the literature (see metabolite annotation details in Supplementary Table S1, Figs S1, S2).

### Pattern recognition and statistics

Metabolite data were log10-transformed without missing value imputation. Pareto-scaling was used for multivariate analyses (Simca P+ 12.0.1, Umetrics, Umeå, Sweden). Principal component analysis (PCA) was performed in order to display general variation and patterns in the metabolite data. Orthogonal projections to latent structures discriminant analysis (OPLS-DA) was performed to inspect which compounds separate ambient and decreased VPD samples. OPLS-DA separates the predictive variation related to VPD treatment from orthogonal variation unrelated to the treatment, such as variation caused by experimental plots. OPLS-DA models were produced separately for birch and aspen data sets. Metabolites with variable importance to projection (VIP) values >1 and p(corr) values <−0.3 or >0.3 were considered relevant in separating the treatments (Supplementary Table S2). A VIP value >1 is a generally accepted limit for a significant effect in OPLS-DA models (Wheelock and Wheelock, 2013). S-plots of two OPLS-DA models were combined to produce shared and unique structure (SUS) plots. SUS plots are used to visualize unique and shared structures between multivariate models, and they are produced by plotting the correlation of the predictive component [p(corr)] of each model with one another (Wiklund *et al.*, 2008). It is common to use this approach for omics data to find metabolite markers associated with specific stress factors [herbivory, Marti *et al.* (2013); water stress, Warren *et al.* (2012); wounding, Boccard and Rutledge. (2013)]. Warren *et al.* (2012) employed OPLS-DA and SUS plot analysis to inspect the metabolite changes in response to water stress in two *Eucalyptus* species. Here, the SUS plot was used to compare metabolite changes in response to decreased VPD between the two species, silver birch and hybrid aspen.

Complementary to the multivariate statistics, the effect of decreased VPD on the means of metabolite levels, leaf traits, and mineral nutrient concentrations was tested using a nested ANOVA model (IBM SPSS Statistics 19), where the experimental plot was a random factor nested within VPD treatment (fixed factor). Ranked values were used for statistical analysis of mineral nutrient data (non-parametric nested ANOVA model). False discovery rate (FDR) correction for multiple analysis was conducted for individual metabolite levels, a *q*-value <0.05 being considered significant (Benjamini and Hochberg, 1995).

## Results

### Leaf traits and mineral nutrient content of leaves

**Fig. 1.**
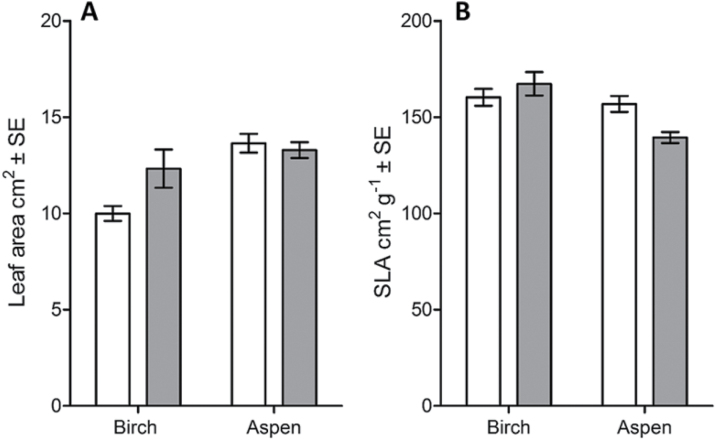
Leaf area (A) and SLA (B) of birch and aspen. Data are represented as mean ±SE (*n*=14–17), white bars represent ambient VPD (control) and grey bars decreased VPD (humidification). The effect of VPD was tested with nested ANOVA (*P<0.05, **P<0.01).

Leaf area and SLA varied between experimental plots, and were not affected by VPD conditions (Fig. 1). In birch and aspen leaves, Na concentration was higher, and the K:Na ratio was subsequently lower, in decreased VPD than in ambient VPD (Table 1). Other nutrients were not affected by VPD treatment in birch leaves (Table 1). In aspen leaves, the N concentration and N:P ratio were lower and concentrations of Ca, Mg, P, Zn, Fe, and V were higher in decreased VPD than in ambient VPD (Table 1).

**Table 1.** Foliar mineral nutrient concentrations

	**Birch**			**Aspen**		
	**Ambient VPD**	**Decreased VPD**	**VPD**	**Ambient VPD**	**Decreased VPD**	**VPD**
**Major (mg g** ^**–1**^)						
N	20.7±0.53	22.1±1.03		21.8±0.30	20.9±0.33	*
Ca	10.0±0.50	9.3±0.37		19.7±1.95	25.3±1.38	*
K	7.4±0.32	8.8±1.31		5.7±0.14	5.3±0.52	
Mg	3.9±0.14	3.8±0.25		3.2±0.21	3.9±0.25	*
P	2.9±0.24	3.2±0.27		1.7±0.04	1.9±0.09	*
S	1.2±0.08	1.4±0.09		1.6±0.09	1.8±0.06	
**Minor (mg g** ^**–1**^)						
Mn	1.08±0.060	1.06±0.112		0.26±0.034	0.32±0.028	
Zn	0.24±0.026	0.28±0.028		0.28±0.049	0.40±0.016	*
Fe	0.07±0.004	0.14±0.043		0.06±0.003	0.09±0.002	**
Na	0.08±0.013	0.73±0.314	**	0.05±0.010	0.32±0.100	*
B	0.02±0.001	0.02±0.002		0.02±0.002	0.03±0.003	
**Trace (µg g** ^**–1**^)						
V	11.2±0.64	13.9±1.29		9.3±0.70	12.5±1.06	*
Cu	4.8±0.29	5.7±0.45		6.0±0.34	6.3±0.44	
Ni	2.0±0.32	2.0±0.17		1.8±0.20	1.3±0.13	
Cr	0.9±0.08	1.5±0.31		2.0±0.59	1.6±0.28	
Cd	0.3±0.02	0.3±0.03		1.1±0.19	1.6±0.15	
Co	ND	ND		0.4±0.07	0.6±0.12	
**Ratio**						
N:P	7.5±0.72	7.6±0.77		12.6±0.40	11.3±0.68	*
K:Na	106.3±15.85	22.0±5.40	**	152.7±39.68	22.1±3.65	**

Statistically significant effects for VPD were tested by nested ANOVA (**P<0.01, *P<0.05).

Data are represented as mean ±SE, *n*=6, ND, not detected.

### Metabolite responses to decreased VPD

**Fig. 2.**
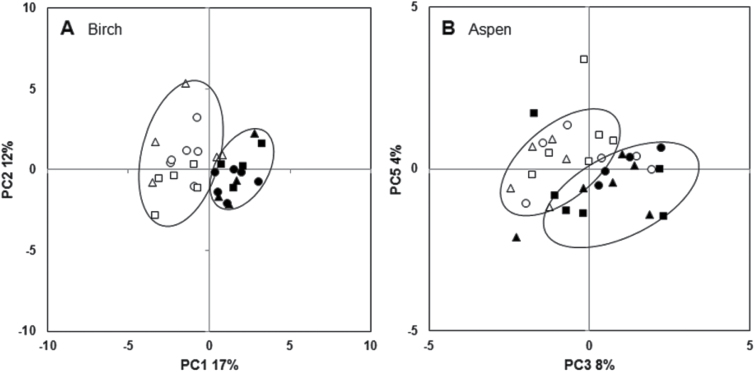
Principal component analysis (PCA) of metabolic profiles in ambient VPD and decreased VPD. PCA of GC-MS metabolites and starch showed that ambient VPD (*n*=16) and decreased VPD (*n*=14) samples of birch formed distinct groups on the basis of the first component, explaining 17% of the variation (A). Ambient VPD (*n*=17) and decreased VPD (*n*=16) samples of aspen formed groups on the basis of the third (8%) and fifth (4%) components (B). Open symbols represent ambient VPD (control) samples and filled symbols decreased VPD (humidification) samples; different symbols represent the three replicate experimental plots.

In birch leaves, 28 metabolites showed significant differences in their means between ambient VPD and decreased VPD samples by nested ANOVA (16 metabolites significant after FDR correction) (Supplementary Table S1). In the aspen leaves, levels of six metabolites showed significant VPD treatment effect by nested ANOVA (one metabolite after FDR correction) (Supplementary Table S1). Although there were few significant treatment effects on the individual metabolite means, PCA showed that decreased VPD caused a shift in the foliar metabolite profiles in both species (Fig. 2). Based on the PCA, ambient VPD and decreased VPD samples of birch showed clear separation by the first component, explaining 17% of the variation (Fig. 2A), whereas ambient VPD and decreased VPD samples of aspen leaves were separated by the third (8%) and fifth (4%) components (Fig. 2B).

**Fig. 3.**
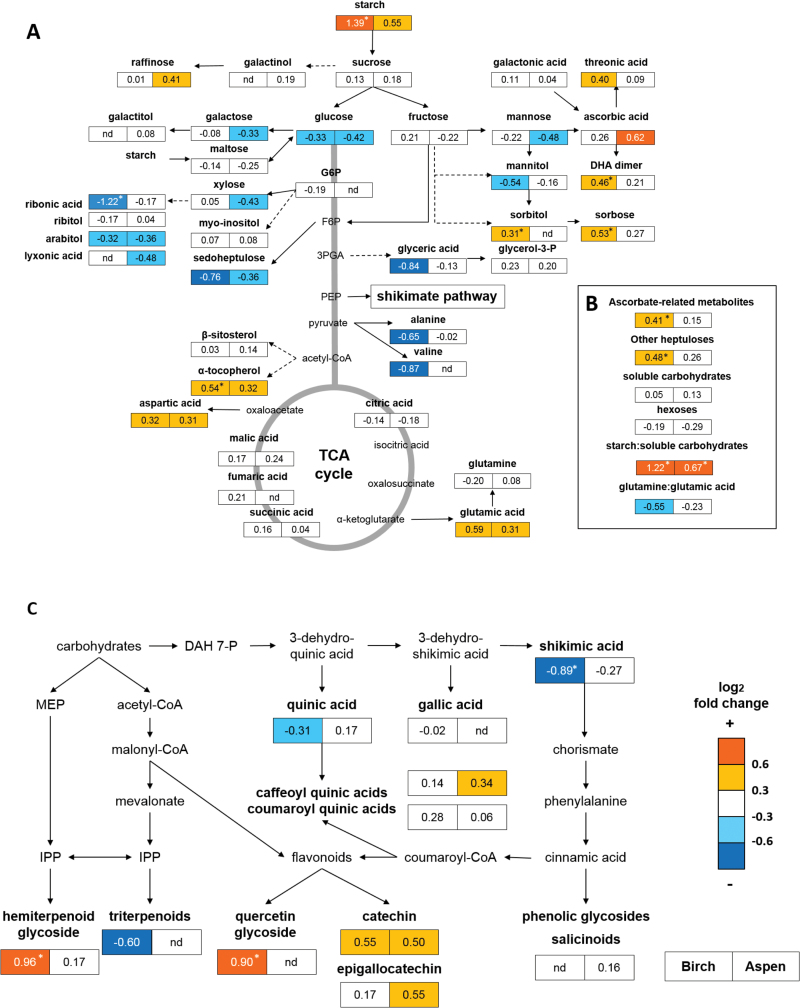
Impact of decreased VPD on primary (A, B) and secondary metabolism (C). Fold changes (log_2_) in the metabolite levels between ambient VPD (control) samples and decreased VPD (humidification) samples in birch (left) and aspen (right) are presented next to the metabolites (nd, not detected). Metabolites that were detected in the GC-MS analysis are in bold. Effects of VPD and experimental plots were tested using the nested ANOVA model. Significant VPD effect is presented next to the fold change (*P<0.05). *n*=11–12 for starch; for GC-MS metabolites, see details in Supplementary Table S1. Gln:Glu, glutamine to glutamate ratio; DHA, dehydroascorbic acid; G6P, glucose 6-phosphate; F6P, fructose 6-phosphate; 3PGA, 3-phosphoglycerate; PEP, phoshoenolpyruvate; DAH 7-P, 3-deoxy-d-arabino-heptulosonic acid 7-phosphate; MEP methylerythritol phosphate; IPP, isopentenyl diphosphate. (This figure is available in colour at *JXB* online.)

Discriminant analysis (OPLS-DA) showed significant separation between treatments for both birch and aspen (model diagnostics in Supplementary Table S3). Based on OPLS-DA, a strong correlation to VPD treatment was observed in relation to carbohydrates, amino acids, ascorbate metabolites, and secondary metabolites (Supplementary Table S2). In birch, the levels of starch, sorbose, sorbitol, quercetin glycoside, heptuloses, α-tocopherol, and hemiterpenoid glycoside were higher, and the levels of shikimic acid and ribonic acid were lower in leaves that were exposed to decreased VPD than to ambient VPD (nested ANOVA) (Fig. 3). The total pool of ascorbate-related metabolites (sum of AsA, dehydroascorbic acid dimer, diketogulonate, threonic acid, and threonolactone) was higher in decreased VPD than in ambient VPD in birch leaves (Fig. 3). In addition to the aforementioned metabolites, phenolic compounds, triterpenoids, and unidentified metabolites also contributed to the separation of ambient VPD and decreased VPD samples of birch in multivariate analyses (Supplementary Table S1, S2).

**Fig. 4.**
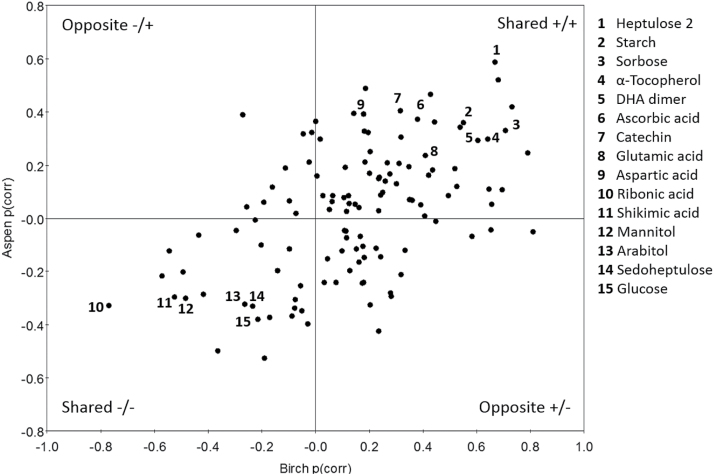
Shared and unique structures (SUS) plot. The plot is constructed by correlating p(corr) values from birch and aspen OPLS-DA models, where p(corr) is the OPLS-DA loading scaled as a correlation coefficient. The levels of metabolites that are located in the upper right quadrant were higher, and the levels of metabolites that are located in the lower right quadrant were lower in decreased VPD than in ambient VPD samples in both species. Metabolites which are located close to the origin, did not show response to the treatment. Metabolites which are located along the axes displayed species-specific responses and metabolites in the upper left or lower right quadrants displayed opposite responses to the treatment in birch and aspen. See Supplementary Fig. S3 for a detailed SUS plot including all metabolite names.

A common response to decreased VPD in both species was high starch content relative to soluble carbohydrates (Fig. 3). Overall, the shift in metabolite profile in response to decreased VPD was highly similar in birch and aspen, although the response was stronger in birch than in aspen leaves (Figs 3, 4). Most of the metabolites displayed similar response patterns in response to decreased VPD, regardless of the species, as shown by SUS plot analysis containing 125 metabolites common in both species (124 GC-MS metabolites and starch) (Fig. 4; Supplementary Fig. S3). For example, the levels of starch, sorbose, heptuloses, glutamic acid, aspartic acid, α-tocopherol, ascorbate-related metabolites, catechin, and five tentative phenolic compounds (2189_204, 2433_204, 2538_217, 2924_361, and 3126_204) were higher (Supplementary Table S1), and the levels of shikimic acid, ribonic acid, arabitol, mannitol, glucose, and sedoheptulose were lower in decreased VPD than in ambient VPD in both species (Figs. 3, 4; Supplementary Fig. S3).

The metabolite responses of silver birch leaves to decreased VPD were partly similar (starch, ribonic acid, glucose, quercetin glycoside, coumaroyl quinic acids, shikimic acid, and amino acids) and partly different (soluble sugars and antioxidants) in field and growth chamber experiments (Supplementary Fig. S4).

## Discussion

### Starch and soluble sugars

As expected, humidification treatment and subsequent decrease in VPD conditions elicited changes in primary and secondary metabolism of tree foliage. The magnitude of metabolite responses to decreased VPD varied between birch and aspen, but the direction of responses was highly similar in both species. As expected, starch production was promoted by decreased VPD in the field, which is in line with the results obtained from growth chamber experiment with young silver birch (Lihavainen *et al.*, accompanying manuscript). Thereby, the total non-structural carbohydrate (NSC) content was high in the tree leaves in decreased VPD. In the chamber experiment, this was not the case; total NSC content was not affected by VPD, because total soluble sugar content declined in low VPD treatment (Lihavainen *et al.*, accompanying manuscript). Nevertheless, a common response to decreased VPD in the growth chamber and in the field, and in both species, was the high starch content relative to soluble sugars.

Starch content of leaves is highly influenced by sink–source dynamics (Sonnewald and Willmitzer, 1992; Paul and Foyer, 2001). If the growth of sink tissues is more inhibited than photosynthesis under stress conditions, reduced carbon can build up in the chloroplasts. When there is a surplus of carbon in the leaves, plants typically allocate it to storage compounds such as starch, which serves as a transient reserve of reduced carbon when sucrose synthesis and/or transport is restricted (Sonnewald and Willmitzer, 1992; Paul and Foyer, 2001). High starch content relative to soluble sugars indicates that carbon storage is favoured over transport, respiration, and growth in decreased VPD. Inhibited growth rate and foliage development implicate that trees were sink limited under decreased VPD (Sellin *et al.*, 2015). Levels of heptulose sugars, other than sedoheptulose, were higher in leaves exposed to decreased VPD than to ambient VPD. The metabolic function of seven-carbon sugars in plants is not well understood, but they have been proposed as factors contributing to the carbon balance of plants, acting as alternative carbon storage and transportable carbohydrates (Häfliger *et al.*, 1999; Liu *et al.*, 2002; Ceusters *et al.*, 2013). Changes in heptulose levels may be related to altered carbon flux through the oxidative pentose phosphate pathway under decreased VPD, which was further supported by the low levels of sedoheptulose and pentose sugars (ribonic acid, arabitol, and xylose) in decreased VPD samples.

Leaf fall was delayed in birch trees, but not in aspen trees under decreased VPD and under elevated atmospheric CO_2_ concentration (Godbold *et al.*, 2014), which suggests that the carbon gain of birch trees was prolonged by decreased VPD. Although the photosynthetic capacity of birch leaves decreased (Sellin *et al.*, 2013), delayed leaf fall and high starch content imply that trees have excess carbon reserve rather than being carbon-limited under decreased VPD. Low VPD is known to promote stomatal opening, facilitating CO_2_ influx to the leaf mesophyll (Fordham *et al.*, 2001; Oren and Pataki, 2001), which can lead to higher carbon gain. High NSC content and reduced N content indicate that growth of trees is more limited by nutrient availability than by carbon availability under decreased VPD (Hoch *et al.*, 2003;Körner *et al.*, 2005; Palacio *et al.*, 2008).

### N metabolism and ion homeostasis

Consistent with our hypotheses, the leaf N concentration of aspen was lower under decreased VPD than under ambient VPD. However, in contrast to our expectations, P concentration of aspen was higher in decreased VPD than in ambient VPD, and P and N concentrations of birch leaves were not affected by the treatment. Regardless of the VPD condition, the N:P ratio of birch leaves was ~7.5, which is below critical N:P values for silver birch (Ågren, 2004). The N:P ratio of aspen leaves was below 14, which is considered to be indicative for N limitation (Koerselman and Meuleman, 1996). Trees were thereby generally N limited in the FAHM field regardless of the VPD condition, and N limitation was elicited by decreased VPD only in aspen. Sellin *et al.* (2015) reported that the humidification treatment affected the leaf N and P concentrations most at the upper canopy level. Since we collected the samples from the middle of the canopy level, the difference in N and P concentrations may be less clear. The lack of response in N or P concentrations in birch foliage in humidification treatment may also be explained by acclimation processes ameliorating nutrient uptake of birch trees. At the end of the fourth growing season, the fine root biomass and the number of root tips was higher in birch trees that were grown under decreased VPD than in those grown under ambient VPD (Godbold *et al.*, 2014; Rosenvald *et al.*, 2014). The ectomycorrhizal community structure of birch roots was shifted towards more hydrophilic fungal taxa in humidified plots (Parts *et al.*, 2013).

Amino acid content and composition have been considered to be good indicators of the N status of plants (Cooper and Clarkson, 1989; Gessler *et al.*,1998: Miller *et al.*, 2008). Major amino acids showed similar patterns in response to decreased VPD in both experiments (Supplementary Fig. S4). The glutamine to glutamate ratio, which is considered to reflect the ammonium assimilation rate (Foyer *et al.*, 2003), was 31–78% lower in decreased/low VPD than in ambient/high VPD in field and chamber experiments (Supplementary Fig. S4).

The high Na concentration and low K:Na ratio of tree leaves indicated altered ion homeostasis as a result of humidification treatment. The Na^+^ content of plants has been reported to increase, while K^+^ often decreases in salt stress, waterlogging, and root hypoxia (Erns, 1990; Milroy *et al.*, 2009; Kreuzwieser and Gessler, 2010; Merchant *et al.*, 2010; Isla *et al.*, 2014). Humidification treatment may cause oxygen depletion in soil due to prolonged soil wetness (Hansen *et al.*, 2013), which may explain the observed ion responses. Since Na^+^ and K^+^ ions have similar physiochemical properties, they compete for uptake to the root symplast and for active binding sites (Amtmann and Sanders, 1999). Although Na^+^ can benefit plants, acting as an osmolyte (Maathuis, 2014), excess of Na^+^ over K^+^ ions can disturb the metabolic processes that depend on K^+^ ions including osmoregulation, photosynthesis, and phloem transport of photosynthates (Amtmann and Armengaud, 2009). Sorbitol is involved in osmotic adjustment and it increases in response to cold, salt, and drought stress (Kaplan *et al.*, 2004; Tari *et al.*, 2010; Li *et al.*, 2012). A higher sorbitol level in birch leaves in decreased VPD than in ambient VPD may provide tolerance against osmotic stress.

### Secondary metabolism and antioxidants

Apart from carbon storage, the balance between photosynthetic carbon assimilation and nutrient metabolism can be achieved by directing excess carbon to secondary metabolites through the methylerythritol phosphate (MEP), mevalonate, or phenylpropanoid pathways. Production of carbon-based secondary metabolites through the phenylpropanoid pathway is typically up-regulated in response to nutrient deficiency (Koricheva *et al.*, 1998; Fritz *et al.*, 2006; Schlüter *et al.*, 2012, 2013) and under elevated atmospheric CO_2_ (Peñuelas *et al.*, 1997; Agrell *et al.*, 2000). A lower level of shikimic acid in the leaves exposed to decreased VPD than to ambient VPD was accompanied by higher levels of phenylpropanoids, which are synthesized via the shikimate pathway from phenylalanine. The levels of quinic acid derivatives, catechins, and phenolic glycosides were higher in the leaves in decreased VPD than in ambient VPD in the field.

In birch leaves, the quercetin glycoside level was higher in decreased VPD than in ambient VPD in the field, which is in agreement with the results obtained from the growth chamber study (Lihavainen *et al.*, accompanying manuscript). In the chamber experiment, the levels of coumaroyl quinic acids responded to N supply in birch saplings, but the levels of quercetin and quercetin glycosides were higher in low VPD than in high VPD, regardless of the N supply (Lihavainen *et al.*, accompanying manuscript). In tomato plants, short-term N limitation increased flavonol glycoside levels, and the levels remained high even after N resupply (Larbat *et al.*, 2012). Flavonol glycoside levels also showed an additive increase to successive N limitations (Larbat *et al.*, 2012).

In addition, the products from the MEP pathway (hemiterpene conjugate and α-tocopherol) were present in higher amounts in decreased VPD than in ambient VPD samples. In contrast, levels of triterpenoids, which are produced via the mevalonate pathway competing for isopentenyl diphosphate and malonyl-CoA with the MEP and flavonoid pathways, were lower in decreased VPD than in ambient VPD, indicating a possible trade-off between pathways. Hemiterpenoid glycosides (HTGs) have been reported to accumulate in Arabidopsis leaves in response to nitrate (NO_3_^–^) deprivation and to increase to a lesser extent in response to root oxidative stress, root damage, and K deficiency (Ward *et al.*, 2011). In all cases, the increase in HTG content in Arabidopsis was associated with low foliar nitrate content and was proposed to play a role in N signalling and as a mechanism of carbon flux overflow under N deprivation (Ward *et al.*, 2011; Beale and Ward, 2013). The level of the tentative hemiterpenoid glycoside was high in birch leaves in decreased VPD, suggesting that this carbon overflow mechanism might be extended to trees. It may also indicate changes in nitrate acquisition of trees; recent evidence shows that compared with control plots, less N is taken up as nitrate in humidified plots (Kupper *et al.*, 2015).

Levels of antioxidants, such as ascorbate metabolites, phenolic compounds, and α-tocopherol, were higher in the tree leaves in decreased VPD than in ambient VPD, indicating defence against oxidative stress. Oxidative stress occurs typically due to unfavourable environmental conditions, such as under nutrient deficiency (Mittler, 2002) and excess soil moisture (Possen *et al.*, 2011; Yang *et al.*, 2011). The high levels of antioxidants, NSCs, and phenolic compounds in the tree leaves under humidification suggest that trees have developed defence against various environmental stress factors. High NSC content can predict better survival of trees after environmental disturbances (Dietze *et al.*, 2014; O’Brien *et al.*, 2014), although trees are prone to lose their carbon reserves if they are severely defoliated. In addition, higher levels of phenolic compounds in the leaves in decreased VPD than in ambient VPD provide chemical defence against pests and pathogens. High NSC content and delayed leaf fall in silver birch under decreased VPD (Godbold *et al.*, 2014) indicate that more carbon may be sequestered in leaf biomass. A high content of NSCs and phenolics in leaf litter has been shown to slow down the decomposition rate (Aerts, 1997; Norby *et al.*, 2001). If these chemical attributes persist in leaf litter, increasing air humidity may affect carbon and nutrient cycling and eventually the primary production of the northern forest ecosystems.

### Conclusion

A long-term decrease in VPD caused a shift in foliar metabolite profiles of silver birch and hybrid aspen. The shift in foliar metabolite profiles in response to decreased VPD was for the most part similar in birch and aspen, but stronger in birch. VPD treatment modified the mineral nutrient composition of leaves, implying that long-term exposure to decreased VPD affects nutrient acquisition and ion homeostasis of trees. Decreased VPD promoted carbon storage as starch and carbon allocation to specific primary (heptuloses and sorbitol) and secondary metabolites (flavonoids, hemiterpenoid, and phenolic glycosides). Higher levels of antioxidants in decreased VPD than in ambient VPD indicated protection against oxidative stress.

Among the main climatic factors comprising the global climate change, air humidity has been the least studied in the field of plant biology. This study, performed in realistic field conditions, demonstrates that long-term exposure to decreased VPD conditions affects the foliar metabolic homeostasis in northern trees. Such changes may have important consequences for resistance against environmental stresses that may cascade throughout the entire forest community. In climate change scenarios predicting the consequences for carbon and nutrient dynamics of northern forest ecosystems, air humidity should thus be considered as an important factor.

## Supplementary data

Supplementary data are available at *JXB* online.

Table S1. Results of GC-MS analysis and annotation details of foliar metabolites of birch and aspen.

Table S2. OPLS-DA VIP (variable importance to projection), p and p(corr) values.

Table S3. OPLS-DA model diagnostics.

Figure S1. Annotation of hemiterpenoid glycoside by GC-MS.

Figure S2. Annotation of salicortin, tremulacin, and tremuloidin based on fragmentation patterns by GC-MS.

Figure S3. Shared and unique structures (SUS) plots including metabolite names.

Figure S4. Impact of decreased VPD on primary and secondary metabolism of silver birch leaves in field and growth chamber experiments.

Supplementary Data

## Abbreviations:

AsA: ascorbic acid
FAHM: free air humidity manipulation
FDR: false discovery rate
HTG: hemiterpenoid glycoside
MEP: methylerythritol phosphate
MSTFA: *N*-methyl-*N*-(trimethylsilyl)trifluoro-acetamide
NSC: non-structural carbohydrate
OPLS-DA: orthogonal projections to latent structures discriminant analysis
PCA: principal component analysis
RH: relative humidity
SLA: specific leaf area
SUS: shared and unique structures in OPLS-DA
TCA: tricarboxylic acid
TMCS: trimethylchlorosilane
VPD: vapour pressure deficit.
