# Supplementary material for: Artificially decreased vapour pressure deficit in field conditions modifies foliar metabolite profiles in birch and aspen
Source: J Exp Bot. 2016 Jun 2;67(14):4367–78. doi: 10.1093/jxb/erw219 (PMC5301936; doi:10.1093/jxb/erw219)
Supplement: Supplementary Data [file supp_67_14_4367__index.html]

Artificially decreased vapour pressure deficit in field conditions modifies foliar metabolite profiles of birch and aspen — Artificially decreased vapour pressure deficit in field conditions modifies foliar metabolite profiles in birch and aspen — Supplementary Data 

# Artificially decreased vapour pressure deficit in field conditions modifies foliar metabolite profiles in birch and aspen

## Supplementary Data

Data files

- supplementary\_tables\_S1\_S2.xlsx - Supplementary Data
- supplementary\_table\_S3\_figures\_S1\_S4.pdf - Supplementary Data
