# Supplementary material for: Artificially decreased vapour pressure deficit in field conditions modifies foliar metabolite profiles in birch and aspen
Source: J Exp Bot. 2016 Jun 2;67(14):4367–78. doi: 10.1093/jxb/erw219 (PMC5301936; doi:10.1093/jxb/erw219)
Supplement: Supplementary Data [file supp_erw219_supplementary_table_S3_figures_S1_S4.pdf]

Metabolite annotation details

Sedoheptulose was annotated based on the standard compound of sedoheptulose anhydride (Extrasynthese, France), which was heated in 1% hydrochloride solution for 2 h at 80 °C, resulting in sedoheptulose formation (Richtmyer and Pratt 1956). Five other heptulose peaks were detected in the samples. Heptulose peaks 2 and 3 as well as 4 and 5 appeared to make pairs by the correlation analysis (data not shown). These pairs are presumably the *syn*- and *anti*-isomers produced by the methoximation of two heptuloses. Four triterpenoids in birch leaves were annotated as dammarane triterpenoids based on their spectra and the characteristic ion m/z 199.

One compound was putatively annotated as hemiterpenoid glycoside (HTG) (Fig. S1) based on the relative retention and the spectrum with a typical ion m/z 157 (Ward *et al.* 2011). Two compounds were tentatively annotated as dihydroxybenzoic acid glycosides based on the typical ions m/z 355 and m/z 370 for dihydroxybenzoic acid. Three major phenolic glycosides (PG) of aspen leaves, salicortin, tremulacin and tremuloidin, were annotated based on their fragmentation patterns (Fig. S2). Metabolite correlation analysis was performed with R (version 2.14.2) to inspect the clustering of metabolites and the correlation of unidentified metabolites with the annotated ones. In aspen leaves, salicin, salicortin, tremulacin and tremuloidin clustered closely together, which supports the annotation results (data not shown).

References:

Richtmyer NK, Pratt JW. 1956. Sedoheptulose—Its Rotation, Reducing Power, Equilibrium with Sedoheptulosan in Acid Solution, and Crystalline Hexaacetate; Also Crystalline 2,7-Anhydro-β-D-altro-heptulofuranose. *Journal of the American Chemical Society* 78, 4717–4721.

Ward J, Baker JM, Llewellyn AM, Hawkins ND, Beale MH. 2011. Metabolomic analysis of *Arabidopsis* reveals hemiterpenoid glycosides as products of a nitrate ion-regulated, carbon flux overflow. *Proceedings of the National Academy of Sciences of the United States of America* 108, 10762–10767.

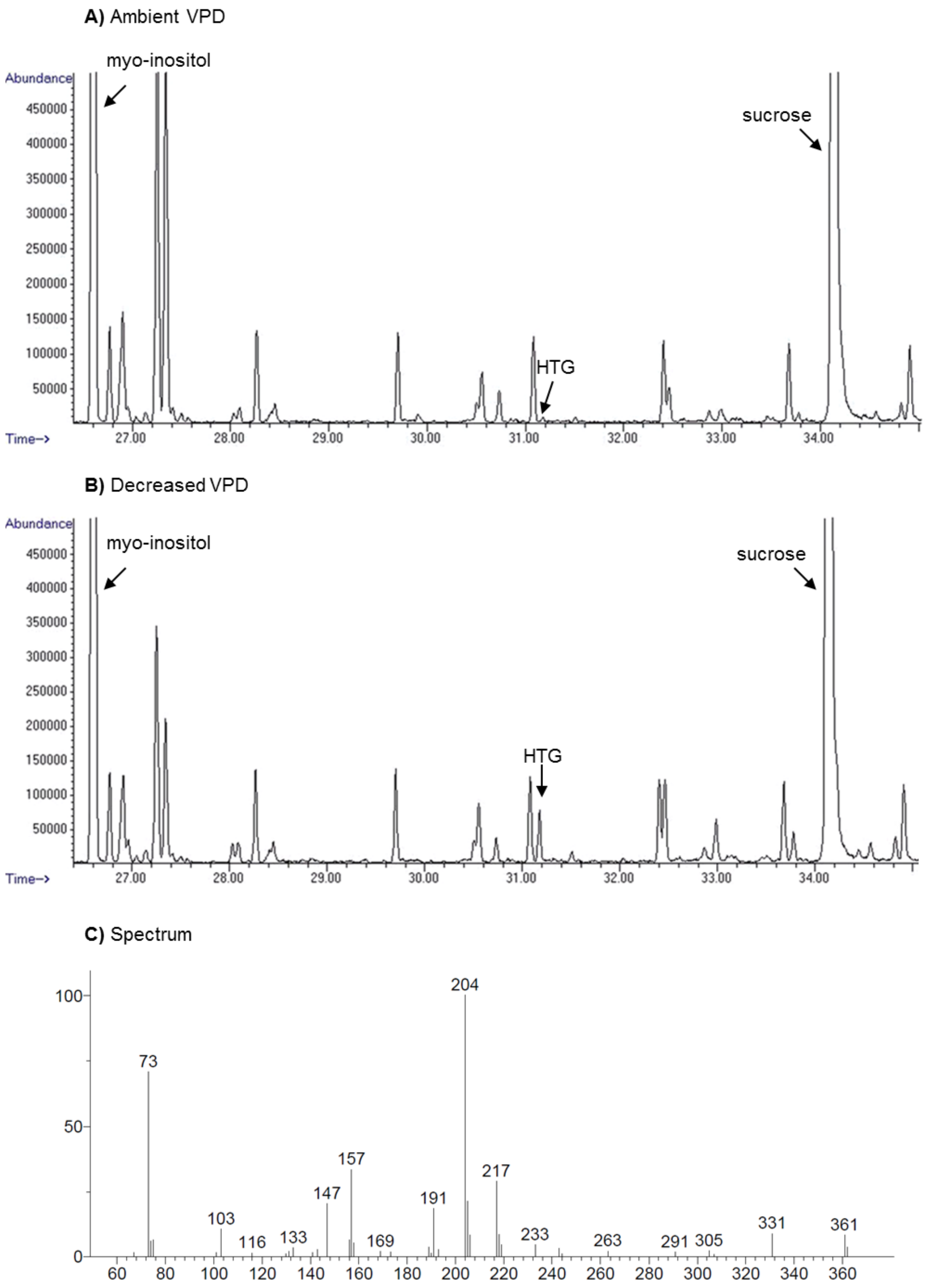

Supplementary Figure S1. Annotation of hemiterpenoid glycoside. Total ion chromatograms of ambient VPD (A) and decreased VPD (B) samples of silver birch leaves show increase in hemiterpenoid glycoside (HTG) peak in response to the humidification treatment. Spectrum shows a typical ion 157 m/z for HTG (C). Relative retention and spectrum matches with the GC-MS analysis of HTG by Ward *et al.* (2011).

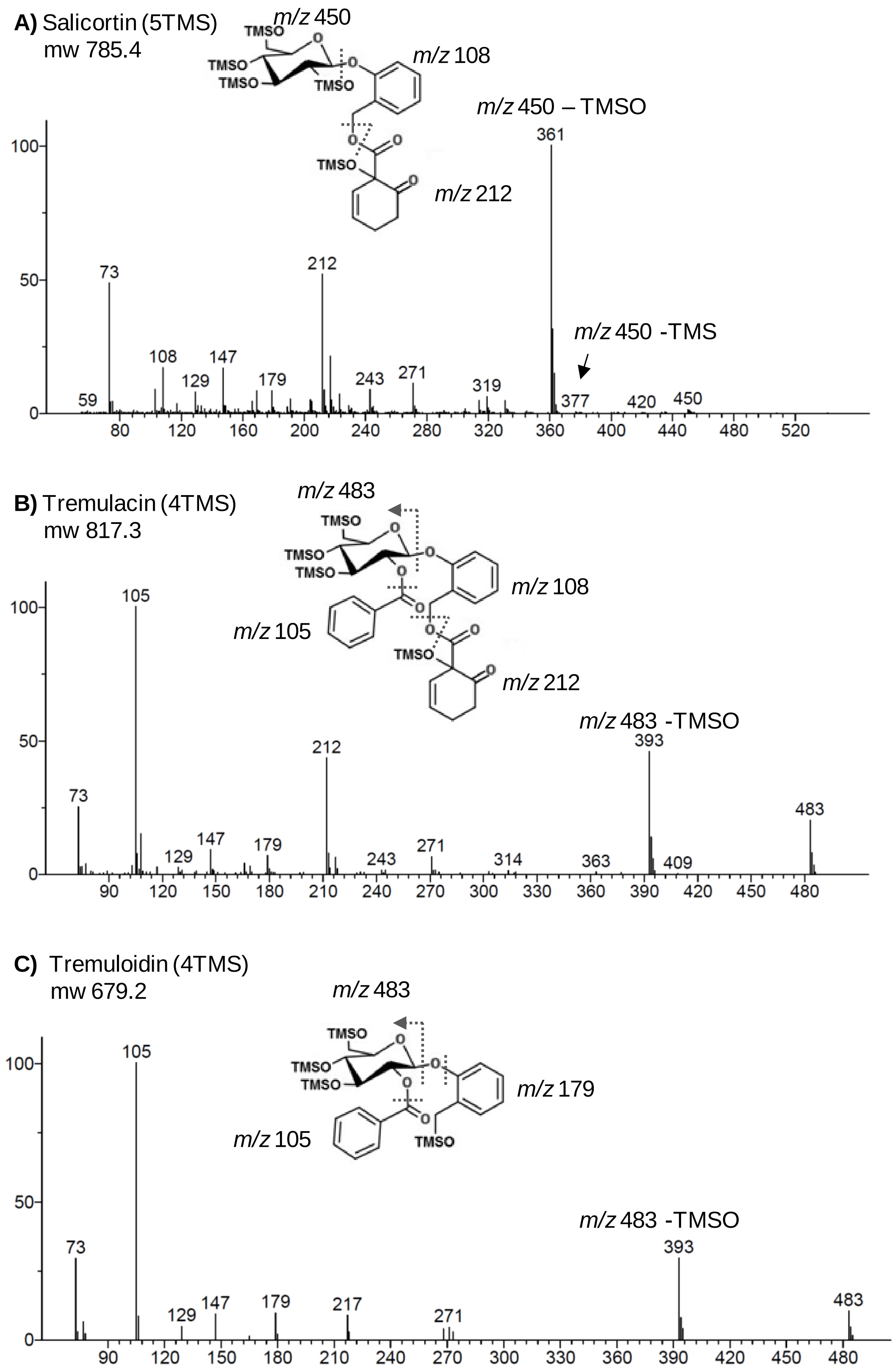

Supplementary Figure S2. Annotation of salicortin (A), tremulacin (B) and tremuloidin (C) based on the fragmentation patterns by GC-MS.

Supplementary Table S3. OPLS-DA model diagnostics. Models were produced separately for metabolite datasets of birch and aspen with Simca-P+. Table shows the number of predictive and orthogonal components, total explained variation by the component(s), fit (R^2Y) and predictivity (Q^2) of the models. The significance of the models was tested by CV-ANOVA. The predictive component explains the variation related to the VPD treatment (humidification) and orthogonal variation is unrelated to the treatment.

**Dataset**	Predictive component	Orthogonal components	R^2Y	Q^2	CV-ANOVA
Birch	1; 13%	2; 13%	0.991	0.853	$p<0.001$
Aspen	1; 6%	3; 53%	0.946	0.594	$p=0.026$

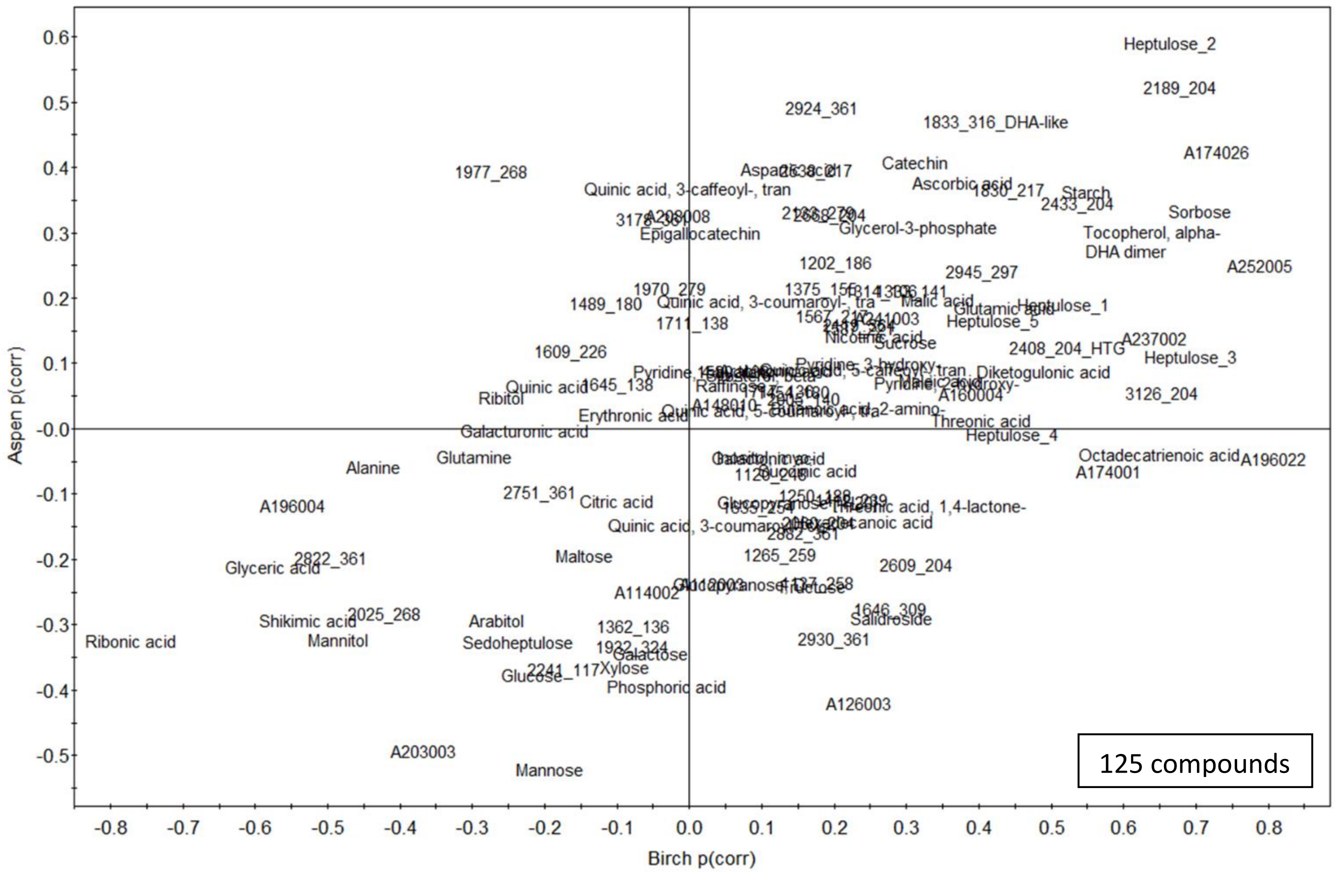

Supplementary Figure S3. Shared and unique structures (SUS)-plots of metabolites. SUS-plots were produced by combining S-plots of two OPLS-DA models (Table S2). The levels of metabolites which are located on the upper right corner of the SUS-plots were higher, and metabolites on the lower left were lower in both species (birch and aspen) in decreased VPD than in ambient VPD. Metabolites, which are located close to origo, did not show response to the treatment. Metabolites which are located along the axes displayed species-specific responses, and metabolites which are located in the upper left or lower right corner displayed opposite responses to the treatment in birch and aspen. p(corr) is the OPLS-DA loading scaled as a correlation coefficient. Most of the leaf metabolites which were detected in both species responded similarly to decreased VPD in field conditions regardless of the species.

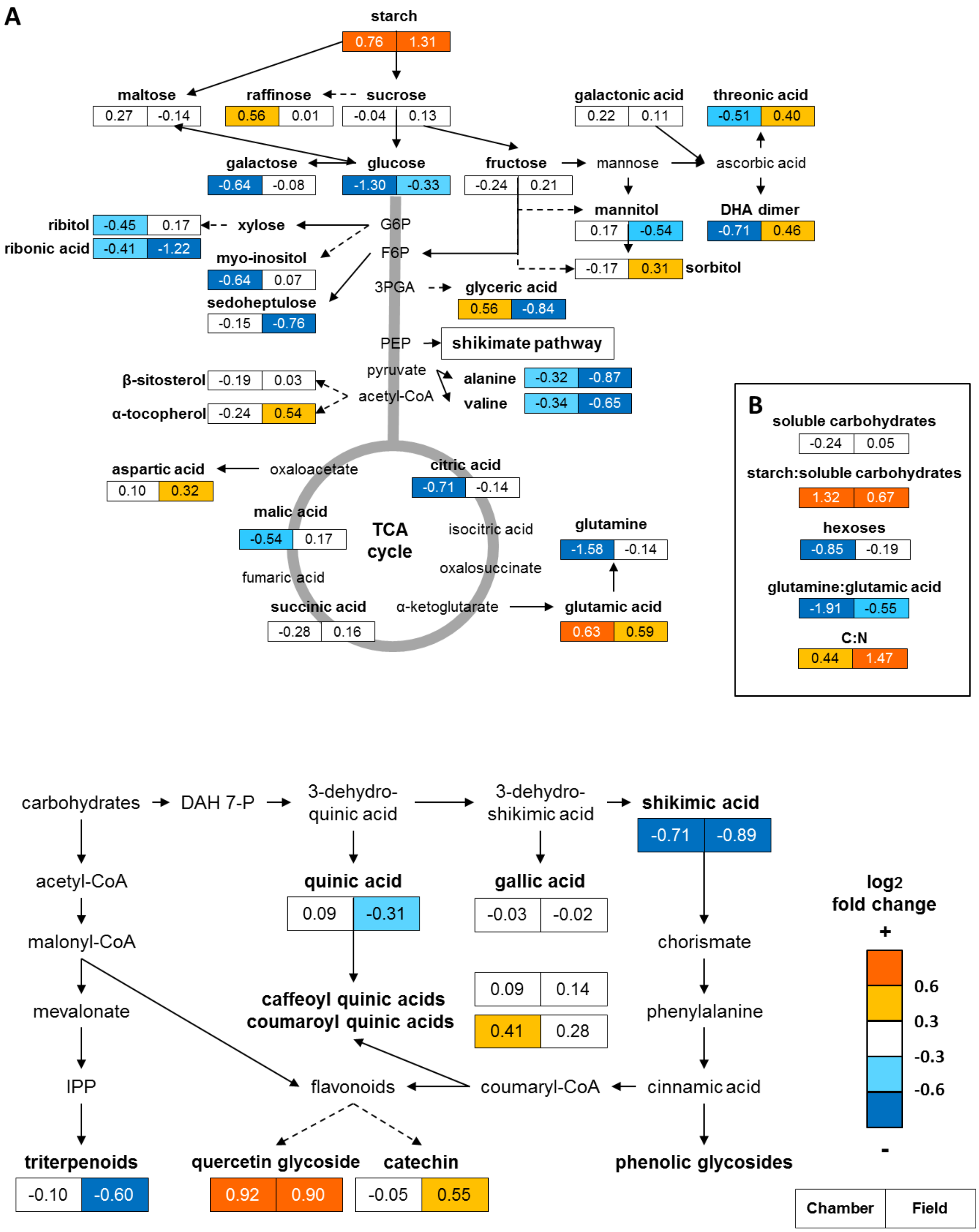

Supplementary Figure S4. Impact of decreased VPD on primary (A) and secondary (B) metabolism of silver birch leaves in field (right) and in growth chamber (left) experiments. Fold changes ($\log_2$) in the metabolite means between ambient/high VPD and decreased/low VPD are represented beside the metabolites. In the field experiment, leaf samples were

collected during the 4th humidification season. In the chamber experiment, samples were collected when plants had been exposed to high VPD (60% RH) and to low VPD (95% RH) for 26 days.
